# Tetrandrine, a Compound Common in Chinese Traditional Medicine, Preferentially Kills Breast Cancer Tumor Initiating Cells (TICs) *In Vitro*

**DOI:** 10.3390/cancers3022274

**Published:** 2011-05-04

**Authors:** Wei Xu, Bisrat G. Debeb, Lara Lacerda, Jessica Li, Wendy A. Woodward

**Affiliations:** Division of Radiation Oncology, University of Texas M.D. Anderson Cancer Center, Houston, TX 77030, USA; E-Mails: weixu@mdanderson.org (W.X.); bgdebeb@mdanderson.org (B.G.D.); lcalvarez@mdanderson.org (L.L.); lili@mdanderson.org (J.L.)

**Keywords:** breast cancer, tetrandrine, cancer stem cells, tumor-initiating cells

## Abstract

Tetrandrine is a bisbenzylisoquinoline alkaloid found in *Stephania tetrandra*, a Chinese medicine commonly used as an anti-inflammatory. It has extensive pharmacological activity, including positive ion channel blockade and inhibition of multiple drug resistance proteins. These activities are very similar to that of salinomycin, a known drug targeting breast cancer initiation cells (TICs). Herein, we tested tetrandrine targeting of breast cancer TICs. SUM-149, an inflammatory breast cancer cell line and SUM-159, a non-inflammatory metaplastic breast cancer cell line were used in these studies. In proliferation assays using 3-(4,5-dimethylthiazol-2-yl)-5-(3-carboxymethoxyphenyl)-2-(4-sulfophenyl)-2H-tetrazolium (MTS), we found that the IC_50_ for inhibition of proliferation is 15.3 ± 4.1 μM for SUM-149 and 24.3 ± 2.1 μM for SUM-159 cells. Tetrandrine also inhibited mammosphere formation, a surrogate for breast cancer TICs growth *in vitro* with IC_50_ around 1 μM for SUM-149 and around 2 μM for SUM-159 cells. Tetrandrine has similar effects on the mammosphere formation from cells isolated from fresh patient sample. Moreover, tetrandrine decreases the aldehyde dehydrogenase (ALDH) positive population in SUM-159 by 45% ± 5.45% P = 0.005. In summary, tetrandrine demonstrates significant efficacy against *in vitro* surrogates for inflammatory and aggressive breast cancer TICs.

## 1. Introduction

The role of tumor-initiating cells (TICs) in carcinogenesis and oncological treatment has become a major focus of cancer research. The cancer stem cell theory posits that only a small, specific population of cells within a tumor has the capacity to recapitulate all of the heterogeneous cell types in a metastatic or locally recurrent tumor. These cells are regulated by developmental pathways critical to normal stem cell survival that are attractive targets to potentially eliminate this population. Using approaches developed in the hematopoietic malignancies, Al-Hajj and colleagues were the first to purify a subpopulation of human breast cancer cells that display tumor initiating properties. As few as 100 of these cells, characterized by the expression of cell surface markers Lin^−^CD44^+^CD24^−/low^, re-generated tumors when orthotopically implanted in immunocompromised mice, and these cells were then considered TICs. In contrast, 20,000 cells with alternate non-TIC phenotypes failed to form tumors [1]. Dontu and colleagues further refined this phenotype demonstrating that as few as 20 cells from the population positive for both Lin^−^CD44^+^CD24^−/low^ phenotype and aldehyde dehydrogenase activity can generate a tumor in immunocompromized mice [2]. Furthermore, Dontu and colleagues adapted the stem cell enriched sphere culture system described in the central nervous system literature to the normal mammary gland and was able to demonstrate *in vitro* self-renewal (the ability to go through numerous cycles of cell division while maintaining the undifferentiated state) and maintenance of multi-lineage differentiation potential in mammosphere culture of normal human breast epithelial cells [3]. In this method, mammary gland cells are cultured in suspension in serum-free, growth factor enriched media. We and others have demonstrated that this approach can be adapted for breast cancer cell lines [4-6]. This approach provides an *in vitro* model to study TICs and develop therapies targeting TICs.

Tetrandrine is a bisbenzylisoquinoline alkaloid found in *Stephania tetrandra*, a Chinese medicine which has been used to treat hypertension and inflammation for thousands of years. Tetrandrine has been shown to have multiple pharmacological activities including immunosupression [7], anti-hypertensive [8] and anti-tumor activity [9]. At the molecular level, tetrandrine was found to block calcium channels [10] and multiple drug resistant proteins (MDR) [11]. Recently, it was reported tetrandrine inhibited β-catenin signaling in a colon cancer xenograft model [12],which is of interest given that β-catenin plays an important role in breast cancer TICs [13,14].

Herein, we screened tetrandrine for efficacy against *in vitro* surrogates of breast cancer TICs. We demonstrate that tetrandrine can inhibit the growth of breast cancer cells. We also found that tetrandrine can inhibit primary mammosphere formation, a surrogate of breast cancer TICs *in vitro* in IC_50_ 10 times lower than the IC_50_ of cell proliferation inhibition. Tetrandrine inhibits the mammosphere formation of cells isolated from fresh patient sample with similar IC_50_. Moreover, we found that tetrandrine can decrease ALDH positive population, which is a putative marker for breast cancer TICs. In summary, tetrandrine can effectively targets *in vitro* breast cancer TIC surrogates.

## 2. Results and Discussion

### 2.1. Tetrandrine Inhibits Proliferation in Breast Cancer Cell Lines

**Figure 1.**
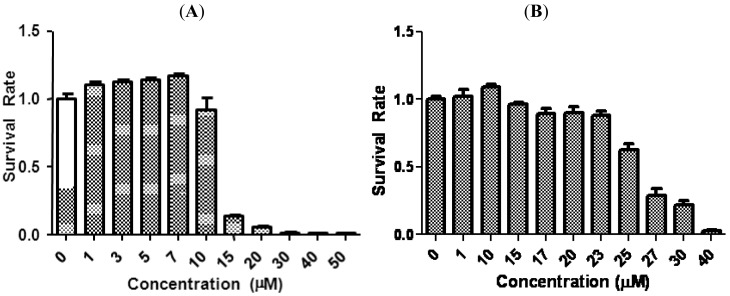
Tetrandrine inhibited proliferation in breast cancer cells. SUM-149 and SUM-159 were treated with increasing concentrations of tetrandrine for 96 h. The anti-proliferation effect of tetrandrine was measured by MTS assay. Error bars represent standard deviation. The data shown are representatives of three independent experiments.

We first evaluated the antiproliferative effects of tetrandrine in two aggressive human breast cancer cell lines, SUM-149 and SUM-159, by MTS assay. Cells were treated with increasing concentrations of tetrandrine for 96 h and the ratio of viable cells of treatment relative to control is plotted in Figure 1. Cell survival decreased as the concentration of tetrandrine increased, with an IC_50_ 15.3 ± 4.1 μM for SUM-149 and 24.3 ± 2.1 μM for SUM-159. These IC_50_ ranges are similar to published results regarding proliferation inhibition in other breast cancer cell lines. For example, IC_50_ for anti-proliferation in MBA-231, a well established breast cancer cell line, is between 10–20 μM [12].

### 2.2. Tetrandrine Inhibits Mammosphere Formation in Breast Cancer Cell Lines

**Figure 2.**
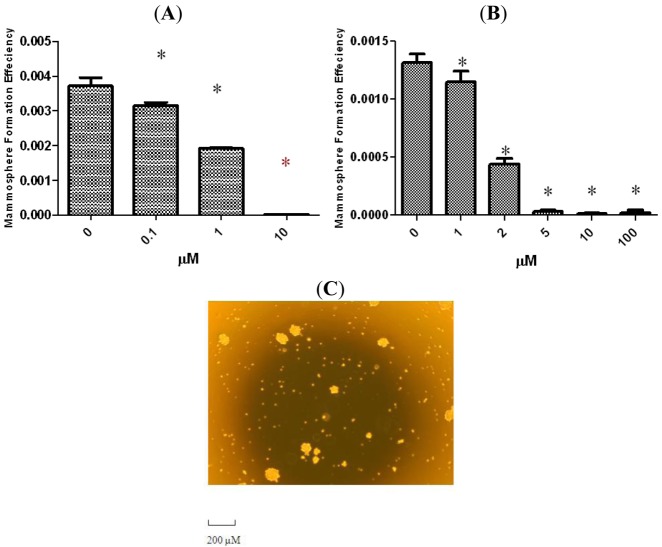
Inhibitory effect of Tetrandrine on mammosphere formation. SUM-149 (**A**) and SUM-159 (**B**) cells were cultured in mammosphere-forming conditions. SUM-149 and SUM-159 cells were incubated with indicated dose of tetrandrine or DMSO in mammosphere formation media for 7 days. Tetrandrine treatment reduced the number of primary mammospheres in a dose dependent manner; (**C**) Representative image of mammospheres from SUM-159. Scale bar = 200 μM. The results are representative of three independent experiments; asterisks indicate P < 0.05 compared to control in all cases. Error bars represent standard deviation.

It has been shown that mammary stem/progenitor cells are enriched in nonadherent spherical clusters of cells, termed mammospheres [3]. When cultured from normal mammoplasty specimens these cells are capable of yielding secondary spheres and differentiating along multiple lineages [3]. From human xenograft tumors radioresistance of sphere cultures correlated with *in vivo* increases in ALDH activity after radiation [15]. To evaluate whether tetrandrine could suppress the formation of mammospheres *in vitro*, we exposed primary SUM-159 and SUM-149 spheres to varying concentrations of tetrandrine. As shown in Figures 2A and 2B, tetrandrine inhibited the formation of primary spheres in the μM range. The IC_50_ for SUM-149 is approximately 1 μM while for SUM-159 it is approximately 2 μM. An interesting note is that IC_50_ for inhibition of mammosphere formation is about 10 times lower than that for proliferation.

### 2.3. Tetrandrine Inhibits Breast Cancer Stem/Progenitor Surrogates Ex Vivo

**Figure 3.**
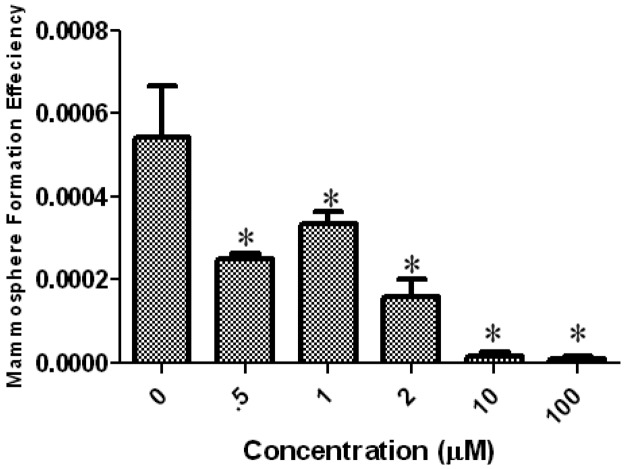
Inhibitory effect of Tetrandrine on mammosphere formation *ex vivo*. Cancer cells purified from fresh patient pleural fluid were seeded in mammosphere media with indicated dose of tetrandrine. In the end, mammospheres were stained with MTT and counted as described. Asterisks indicate P < 0.05 compared to control. Error bars represent standard deviation.

To further confirm the results that tetrandrine inhibits the breast cancer TICs, we used tumor cells purified from fresh patient pleural fluid to test if tetrandrine can inhibit the mammosphere formation in this *ex vivo* model. As shown in Figure 3, tetrandrine inhibited the mammosphere formation in patient sample in the similar dose as that inhibiting cell lines (Figure 3). This result further confirmed that tetrandrine can inhibit the breast cancer TICs.

### 2.4. Tetrandrine Decreases the ALDH Positive Population in Breast Cancer Cells

**Figure 4.**
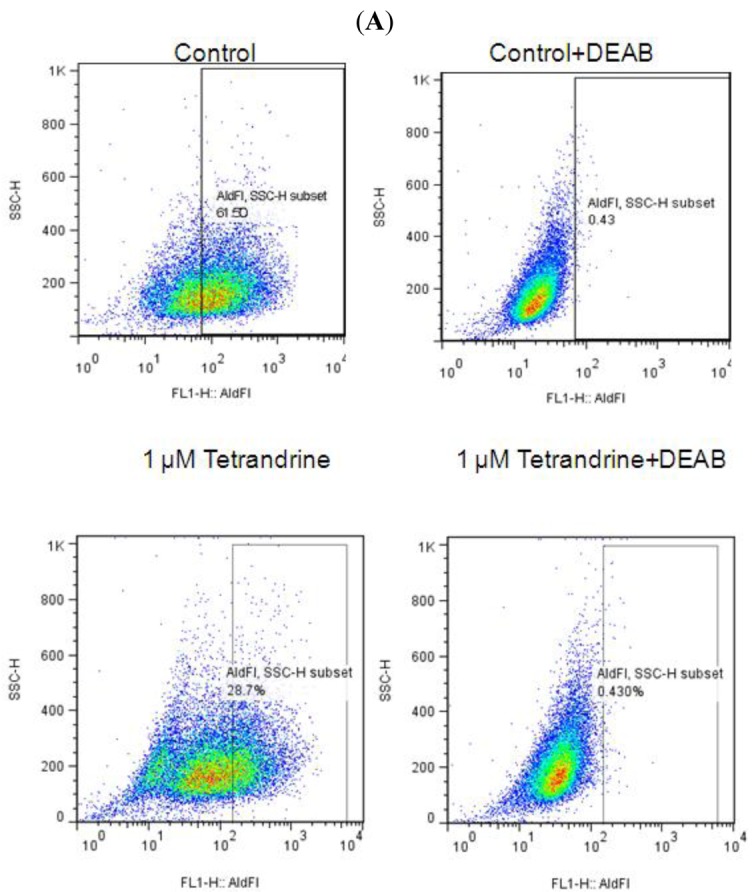

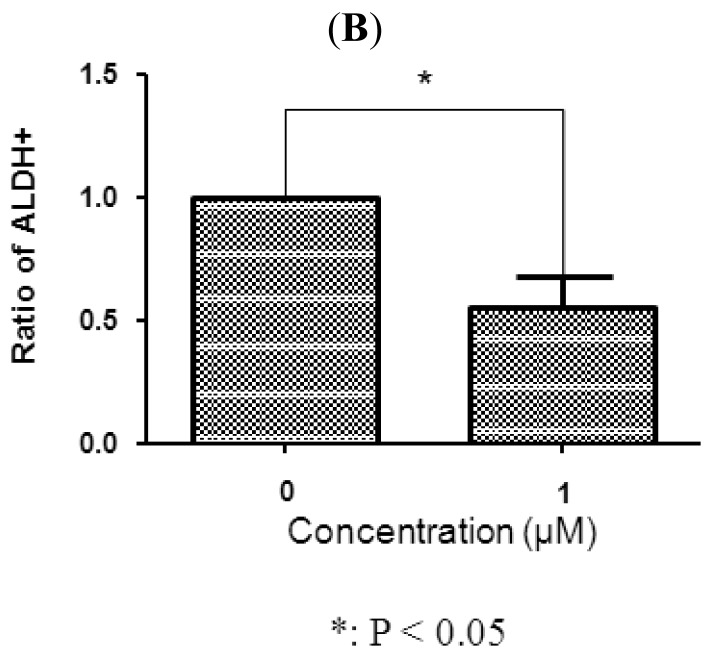
Tetrandrine can decrease ALDH-positive cell population. SUM-159 cells were treated with tetrandrine (1 μM) or vehicle (DMSO) for 4 days and subjected to Aldefluor assay and flow cytometry analysis. (**A**). set of representative flow cytometry dot plots; (**B**). tetrandrine decreased the percentage of ALDH-positive cells (mean of three independent experiments). The error bars represent the standard deviation and Student's one sided t-test was used for comparison.

In breast carcinomas, a cell population with high ALDH activity as assessed by the Aldefluor assay has been shown to enrich for tumorigenic stem/progenitor cells [16]. This cell population is capable of self-renewal and generating tumors resembling the parental tumor [16]. Because SUM-159 has a relatively high percentage of ALDH-positive cells, we selected SUM-159 to examine whether tetrandrine inhibits the tumor-initiating ALDH-positive cells *in vitro*. As shown in Figure 4, 1 μM tetrandrine decreased the ALDH-positive population of SUM159 cells by 42.6% (P < 0.05). Representative flow cytometry dot plots are presented in Figure 4. These data showed that tetrandrine inhibited the ALDH-positive cells at similar concentrations to that inhibiting mammosphere formation and at about 10 fold lower concentration than that inhibiting cancer cells as determined by proliferation assay. This further supports that tetrandrine can specifically target breast cancer TICs.

## 3. Material and Methods

### 3.1. Cell Culture

SUM149 and SUM159 cells were provided by Dr Stephen Ethier (Kramanos Institute, MI, USA) to the Morgan Welch IBC research program and are commercially available (Asterand, Detroit, MI, USA). They were cultured in Ham's F-12 media supplemented with 10% fetal bovine serum (FBS), 1 mg/mL hydrocortisone, 5 mg/mL insulin and 1% antibioticantimycotic. In all experiments, cells were treated with either tetrandrine (Sigma, St. Louis, MO, USA) dissolved in dimethyl sulfoxide (DMSO) (Sigma, St Louis, MO, USA) or DMSO alone.

### 3.2. Proliferation Assay

Cells were plated in 96 cell dish with a specified cell number (5000 cells/well for SUM-159 and 10000 cells/well for SUM-149). Different doses of tetrandrine were added at time zero. After 96 h, media was replaced by a fresh media mixed with 20% MTS (Promega, Madison, WI, USA). After 2 h, the absorbance at 490 nm is determined in a 96 well platereader (PerkinElmer, Waltham, MA, USA).

### 3.3. Mammosphere Assay

To generate mammospheres, cells were grown in serum-free MEM supplemented with 20 ng/mL bFGF, 20 ng/mL EGF and 2% B27 (all from Invitrogen, Carlsbad, CA, USA). Cells were plated at 10,000–20000 cells/ml as indicated and grown for 7 days. Sometimes, drug (tetrandrine) with indicated concentration was added to the media in the beginning. In the end of experiments, spheres were visualized with 3-(4,5-dimethylthiazol-2-yl)-2,5-diphenyltetrazolium bromide (MTT) (Sigma, St. Louis, MO, USA) and counted with an automated colony counter (Oxford Optronix, Oxford, UK).

### 3.4. Aldfluor Assay

The Aldefluor assay was carried out following the manufacturer's guidelines (StemCell Technologies, Vancouver, Canada). Briefly, SUM-159 cells were seeded in conventional adherent media as described. After 24 h of seeding, cells were treated with 1 μM tetrandrine for 96 h. In the end, single cells obtained from cell cultures were incubated in an Aldefluor assay buffer containing an ALDH substrate, bodipy-aminoacetaldehyde (1 μmol/L per 1,000,000 cells), at 37 °C for 30 min. As a negative control, half of the cells from each sample was incubated under identical condition in the presence of the ALDH inhibitor diethylaminobenzaldehyde (DEAB). After incubation, cells were washed with Aldefluor assay buffer and analyzed by flow cytometry to measure ALDH-positive cell population.

### 3.5. Isolating Tumor Cells from Patient Pleural Fluid

Cells from patient pleural fluid were obtained on a clinical protocol approved by the institutional review board from a patient with metastatic breast cancer (IBC). Briefly, the pleural fluid was centrifuged at 400 g for 30 min. The pellets were suspended in PBS/HBSS and filtered through 40 μM cell strainer (Invitrogen, Carlsbad, CA, USA). The suspended cells were added to the top of 12.5 Ficoll Histopaque solution (Sigma, St. Louis, MO, USA) and the mix was spun at 2000 rpm for 30 min. The centrifuged cells were washed by PBS 3 times and suspended in PBS for further usage, such as mammosphere formation assay.

### 3.6. Statistics

IC50 was calculated for each proliferation replicate and the result presented as an average +/−standard deviation. Error bars represent standard deviation in the figures. Student's one-sided *t*-test was used for comparison of all continuous data and P < 0.05 considered significant.

## 4. Conclusions

More and more evidence suggests that TICs sustain cancer growth and cause tumor metastasis and recurrence after therapies. From a clinical point of view, therapies targeting TICs may have the potential to cure the cancer because they target the root of tumor. However, more and more evidence shows that TICs are more resistant than non-TICs to multiple cancer therapies including chemotherapy and radiation [6,17-20]. Herein we demonstrate that Tetrandrine, a well-tolerated oral agent found in a common Chinese medical compound is active against *in vitro* surrogates for TICs in cell lines derived from the most aggressive breast cancers and cells purified from fresh patient samples.

In several solid tumor types, radiation has been shown to enrich for TICs by selectively targeting the non-TICs [6,17,18]. In clonogenic assays comparing first and second generation tumorspheres from MCF-7 breast cancer cells, the TIC enriched second generation spheres are more resistant to radiation [19] and in our laboratory, similar to the work reported by Phillips *et al.* [6], we find increased radiation resistance in TIC enriched tumorspheres compared to more differentiated colonies formed from monolayer culture in all lines tested independent of the molecular phenotype (MCF-7, SUM-190, SUM-149, MDA-MB-231 and short term primary human breast cancer cells) [6,19]. Numerous studies across other tumor sites have also reported intrinsic radiation resistance in TICs [20]. TICs are also resistant to chemotherapies. Li *et al* found that chemotherapy can increase the percentage of a cell population with CD44^high^CD24^low^ cell surface mark, the putative mark for breast cancer TICs in pre and post treatment patient samples [21,22]. Tanei *et al.* reported that ALDH positivity better distinguishes chemo-resistance compared to CD44^high^CD24^low^ [23].

Much effort has been made to identify compounds that specifically target TICs. Overall, few compounds have been identified to target breast cancer TICs. In one paper, salinomycin, a potassium channel blocker used for treatment of Coccidia parasites in chicken, was identified to target breast cancer stem cells [24]. In another paper, dietary supplement sulforaphane was shown to target breast cancer TICs through inhibition of β-catenin signaling [25]. Because β-catenin signaling has been shown to play an important role in breast cancer TICs self-renewal [13,14] and tetrandrine Tetrandrine, a component of the Chinese traditional medicine compound *Stephania tetrandra*, was found to inhibit β-catenin signaling [12], we used *in vitro* systems to test if tetrandrine can target breast cancer TICs.

Tetrandrine was shown to cause human leukemia cells U937 to go through apoptosis in a mechanism unrelated to calcium channel blockage [26]. Tetrandrine was also reported to target colon cancer. In one case, tetrandrine caused early G-1 arrest in colon cancer cells by induction of G1-S-specific cyclin-dependent kinases [27]. In another case, tetrandrine inhibited the proliferation of colon cancer cells *in vitro* and caused tumor growth delay of colon cancer cell xenografts *in vivo* [12]. Other cancers reported to be sensitive to tetrandrine including liver cancer [28,29] and glioma [30,31]. Tetrandrine has been reported to sensitize various cancers, including neuroblastoma [32] and glioblastoma [33], to radiation therapy. Moreover, because tetrandrine is a MDR protein inhibitor [11], it has been combined with chemotherapeutic agents such as doxorubicin or vincristine to increase the efficacy [34]. MDR1 is the first member of MDR family which is non-specific ion pumps on membranes used by cancer cells to efflux chemotherapeutic compounds and cause chemotherapy resistance. Beyond its original function, over-expression of MDR1 was found to increase hematopoetic stem cells *in vitro* and *in vivo* [35]. Interestingly, salinomycin, the most efficacious drug found to target breast cancer TICs in one chemical library screen [24], was also a MDR inhibitor [36].

Several methods have been developed to isolate and characterize breast cancer TICs *in vitro*. One is mammosphere formation described by Dontu *et al.* [3] and adapted for breast cancer cell lines. Another method is to use cell surface marker expression to distinguish breast cancer TICs from differentiated cells based on tumor outgrowth after transplantation into the cleared mammary fatpads of immunocompromised mice. Both Lin^−^CD24^low^CD44^high^ and ALDH positive have been explored as markers for human breast cancers. Herein, we chose ALDH positive as mark for breast cancer TICs and compared the effect of tetrandrine on proliferation *versus* TIC surrogates, mammosphere forming efficiency and ALDH activity. We found that tetrandrine inhibits proliferation and TIC surrogates and that the inhibition of breast cancer TICs occurs at an IC_50_ about 10 times lower than the dose needed to inhibit the cell proliferation, suggesting specificity of tetrandrine against the breast cancer TICs. Of course the gold standard for TIC studies remains *in vivo* limiting dilution assays and our data are limited by the caveats inherent in all *in vitro* studies. Nevertheless, *in vitro* screening assays are important in selecting agents for further testing, and prior work suggests correlation between *in vitro* TIC surrogates and *in vivo* tumor initiation [15,24]. In addition, activity against primary human breast cancer mammospheres is similar to results obtained from mammosphere assays using immortalized cell lines, which highlights the potential clinical relevance.

Like many chemotherapy agents, tetrandrine has numerous biologic effects, and no specific mechanism has been identified which accounts for the anti-TIC activity. Others have demonstrated inhibition of an important breast cancer TIC mediator, β-catenin, however, tetrandrine failed to decrease the expression of β-catenin in breast cancer cells (data not shown). Further functional signaling analysis, using TOP-GFP a fluorescent β-catenin signaling readout [37], is necessary to rigorously examine this question.

In conclusion, we have shown that tetrandrine can target breast cancer TICs *in vitro* by the mammosphere formation assay and ALDH assay. Further *in vivo* and mechanistic studies assays are needed to fully determine the significance of this finding. Nevertheless, tetrandrine has been used for treatment of hypertension and inflammation in China for thousands of years. Recently, tetrandrine has been tried in clinic to treat refractory and relapsed acute myelogenous leukemia in China [38] and in studies in the United States, tetrandrine has been tested for toxicity in primates [39]. If further studies remain promising, clinical testing alone or in combination with standard chemotherapy may be feasible for this non-toxic orally available drug.
